# Ventilation in patients with intra-abdominal hypertension: what every critical care physician needs to know

**DOI:** 10.1186/s13613-019-0522-y

**Published:** 2019-04-25

**Authors:** Adrian Regli, Paolo Pelosi, Manu L. N. G. Malbrain

**Affiliations:** 1 Department of Intensive Care, Fiona Stanley Hospital, Murdoch Drive, Murdoch, WA 6152 Australia; 2 Medical School, Division of Emergency Medicine, The University of Western Australia, Sterling Highway, Crawley, Perth, WA 6009 Australia; 3 Medical School, The Notre Dame University, Henry Road, Fremantle, Perth, WA 6959 Australia; 4 Department of Surgical Sciences and Integrated Diagnostics, University of Genoa, Genoa, Italy; 5 San Martino Policlinico Hospital, IRCCS for Oncology and Neurosciences, Genoa, Italy; 6 Intensive Care Unit, University Hospital Brussels (UZB), Jette, Belgium; 7 Faculty of Medicine and Pharmacy, Vrije Universiteit Brussel (VUB), Brussels, Belgium

**Keywords:** Intra-abdominal pressure, Intra-abdominal hypertension, Abdominal compartment syndrome, Mechanical ventilation, Recruitment, Compliance, Positive end-expiratory pressure, Ventilator-induced lung injury, Protective ventilation, Driving pressure

## Abstract

The incidence of intra-abdominal hypertension (IAH) is high and still underappreciated by critical care physicians throughout the world. One in four to one in three patients will have IAH on admission, while one out of two will develop IAH within the first week of Intensive Care Unit stay. IAH is associated with high morbidity and mortality. Although considerable progress has been made over the past decades, some important questions remain regarding the optimal ventilation management in patients with IAH. An important first step is to measure intra-abdominal pressure (IAP). If IAH (IAP > 12 mmHg) is present, medical therapies should be initiated to reduce IAP as small reductions in intra-abdominal volume can significantly reduce IAP and airway pressures. Protective lung ventilation with low tidal volumes in patients with respiratory failure and IAH is important. Abdominal-thoracic pressure transmission is around 50%. In patients with IAH, higher positive end-expiratory pressure (PEEP) levels are often required to avoid alveolar collapse but the optimal PEEP in these patients is still unknown. During recruitment manoeuvres, higher opening pressures may be required while closely monitoring oxygenation and the haemodynamic response. During lung-protective ventilation, whilst keeping driving pressures within safe limits, higher plateau pressures than normally considered might be acceptable. Monitoring of the respiratory function and adapting the ventilatory settings during anaesthesia and critical care are of great importance. This review will focus on how to deal with the respiratory derangements in critically ill patients with IAH.

## Background

Intra-abdominal hypertension (IAH) is defined as a sustained increase in intra-abdominal pressure (IAP) equal to or above 12 mmHg [1]. Critical care physicians around the world still underestimate the high incidence of IAH which is around 25% in mixed ICU patients [2, 3].

IAH is associated with increased morbidity and mortality [2, 4] and is mainly caused by too much intra-abdominal volume within the abdominal cavity [5, 6].

**Fig. 1:**
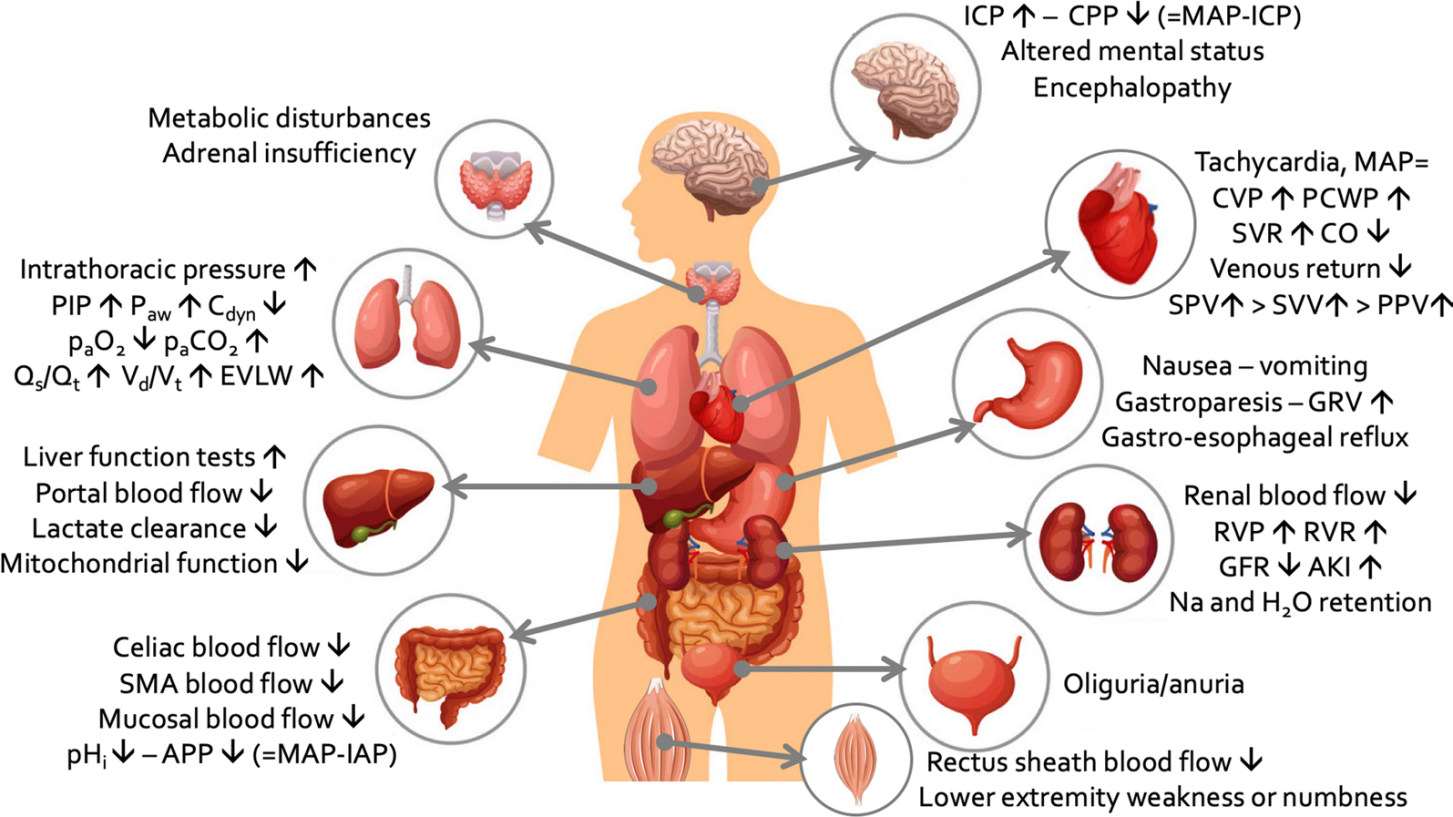
Summary of the most important pathophysiologic effects of increased intra-abdominal pressure on end-organ function within and outside the abdominal cavity. *AKI* acute kidney injury, *APP* abdominal perfusion pressure, *Cdyn* dynamic respiratory compliance, *CO* cardiac output, *CPP* cerebral perfusion pressure, *CVP* central venous pressure, *EVLW* extravascular lung water, *GFR* glomerular filtration rate, *GRV* gastric residual volume, *HR* heart rate, *IAP* intra-abdominal pressure, *ICP* intra-cranial pressure, *ITP* intra-thoracic pressure, *MAP* mean arterial pressure, *PIP* peak inspiratory pressure, *Paw* airway pressures, *PCWP* pulmonary capillary wedge pressure, *pHi* intra-mucosal gastric pH, *PPV* pulse pressure variation, *Qs/Qt* shunt fraction, *RVP* renal venous pressure, *RVR* renal vascular resistance, *SMA* superior mesenteric artery, *SPV* systolic pressure variation, *SVR* systemic vascular resistance, *SVV* stroke volume variation, *Vd/Vt* dead-space ventilation. Adapted from Malbrain et al. with permission [126]

IAH directly impacts on organ function of the abdominal organs such as kidney and liver. Furthermore, IAH can affect the function of organs outside the abdominal cavity including the brain, the cardiovascular system and the lungs [7]. Figure 1 summarizes the pathophysiologic effect of IAH on end-organ function.

IAH affects mainly respiratory mechanics and only in part oxygenation. IAH causes a cephalad shift of the diaphragm thereby increasing intra-thoracic pressures and reducing chest wall compliance and lung volumes [8]. Table 1 lists other factors that affect “chest wall” compliance.

**Table 1:** Factors that affect “chest wall” compliance

Direct effect on chest wall
Pleural effusion
Lung transplant
Sternotomy (post-CABG)
Obesity
Fluid overload
Rib fractures
Indirect effect on chest wall—intra-abdominal hypertension (IAH)
Abdominal distension
Ascites
Fluid overload
Obesity

The aim of this review is to discuss the effects of IAH on respiratory function and the ventilatory management of patients with IAH, needless to state that the ventilatory management of patients with IAH has to take into account not only the respiratory effects of IAH but also the consequences of any underlying chronic lung disease or a newly acquired lung injury.

## Epidemiology

### Intra-abdominal hypertension

Around one in four to one in three patients present with IAH on admission to intensive care unit (ICU) while around one in two will develop IAH within the first week of ICU stay [2, 9]. Moreover, one in twenty mixed ICU patients will develop overt abdominal compartment syndrome, a lethal syndrome with a mortality rate above 75% when left untreated [4]. To this day, patients may have unrecognized IAH as awareness is still low [3]. The risk factors for IAH include abdominal surgery, surgery performed in the emergency setting, severe poly-trauma, abdominal trauma, severe haemorrhagic shock, severe burns, severe acute pancreatitis, large volume fluid resuscitation (especially crystalloid) resulting in fluid overload, ileus, and liver dysfunction [10].

### Respiratory failure and intra-abdominal hypertension

Patients receiving mechanical ventilation are more likely to have IAH [11, 12]. Also, patients with respiratory failure PaO_2_/F_i_O_2_ ratio < 300 mmHg, or receiving positive end-expiratory pressure (PEEP) > 10 cmH_2_O or having a peak airway pressure > 28 cmH_2_O are more likely to have IAH [12, 13]. Others did not find an association between mechanical ventilation and IAH [14]. Table 2 lists the respiratory effects induced by IAH.

**Table 2:** Respiratory effects related to increased IAP

1. Effects on respiratory mechanics (Diaphragm elevation)
Intra-thoracic pressure ↑
• Pleural pressure ↑
• Peak airway pressure ↑ (volume controlled)
• Mean airway pressure ↑
• Plateau airway pressure ↑
Respiratory system compliance ↓
• Chest wall compliance ↓
• Lung compliance =
• Lung volumes ↓ (pressure controlled)
Functional residual capacity (FRC) ↓
Compression atelectasis ↑
Pulmonary vascular resistance ↑
Lower inflection point on PV curve ↑
2. Effects on gas exchange (Reduced gas exchange)
Hypercarbia ↑
Oxygenation ↓
• Dead-space ventilation ↑
• Intra-pulmonary shunt ↑
• Ventilation perfusion mismatch ↑
• Alveolar oedema ↑
3. Clinical effects (Difficult weaning)
Oxygen consumption ↑
Metabolic cost and work of breathing ↑
4. Biological effects
Activated lung neutrophils (experimental) ↑
Pulmonary inflammatory infiltration (experimental) ↑

### Key message 1: Epidemiology and IAH

The average incidence of IAH in critically ill patients is around 25–30% on admission, and the cumulative incidence is around 50% during the first week of ICU stay. There is an association between patients having IAH and respiratory failure.

## Effects of intra-abdominal hypertension on respiratory function

### Effect of intra-abdominal hypertension on lung volumes

#### Pathophysiology

IAH causes a cranial shift of the diaphragm, thereby increasing intra-thoracic pressures affecting lung volumes and respiratory mechanics [8]. IAH is associated with reduced lung volumes as shown in many animal experiments [15–19]. Lung volumes decline with increasing degree of IAH [18, 19].

#### Animal data

In pigs, increasing IAP from baseline to 12, 18 and 22 mmHg decreased end-expiratory lung volumes by 30, 46 and 49% respectively [19]. There are insufficient data to know at what level of IAP lung volumes reduce or atelectasis occurs. At least in pigs, lung volumes decline with increasing degree of IAH [18, 19]. For example, Mutoh et al. [20] inflated in piglet an abdominal balloon in small increments and found that end-expiratory lung volumes reduced even after small increases of IAP. Quintel et al. [15] applied in pigs an IAP of 15 mmHg and measured thoracic lung volumes using computer tomography. IAH increased the percentage of atelectatic as well as poorly aerated lungs.

#### Human data

**Fig. 2:**
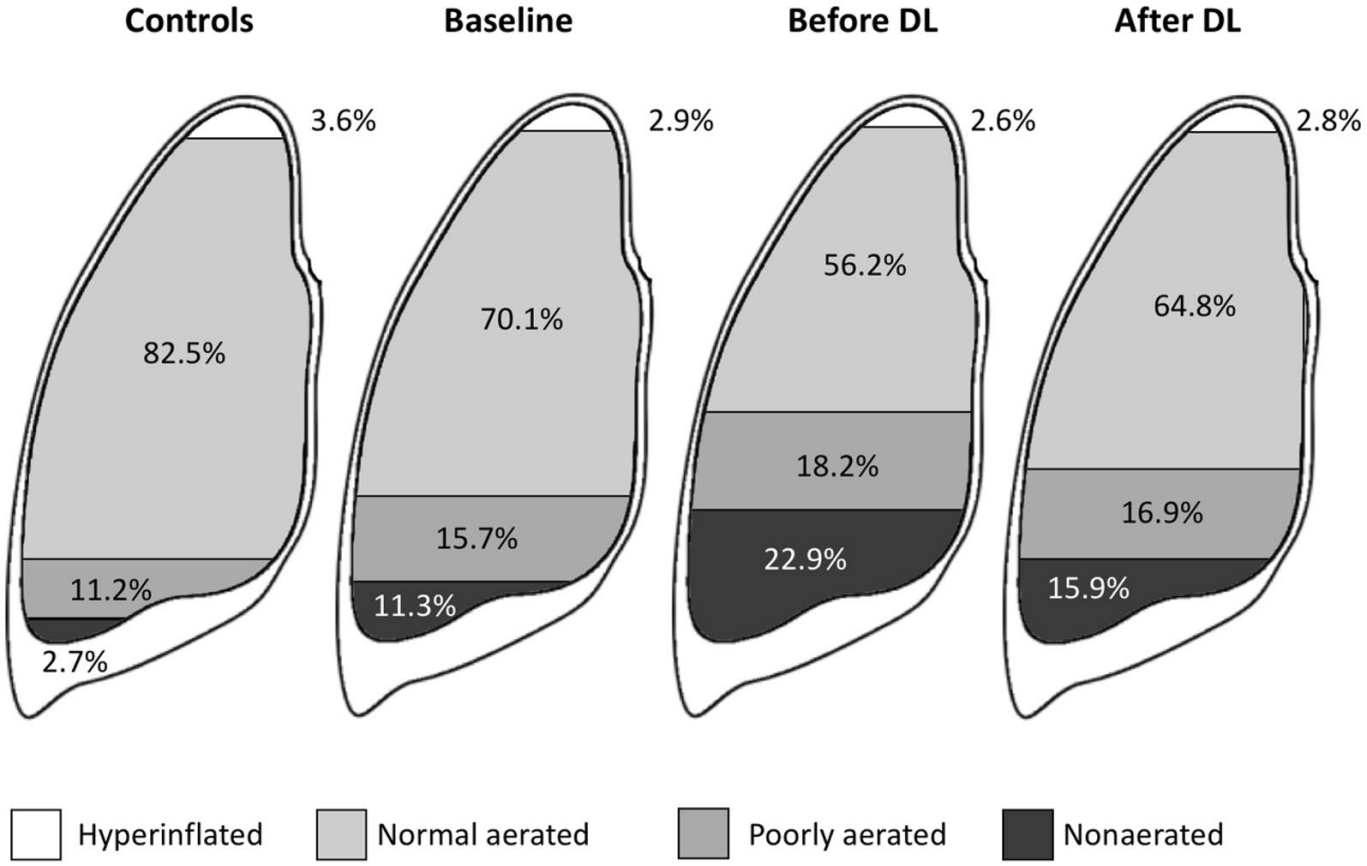
Effect of abdominal hypertension and decompressive laparotomy (DL) on total lung volumes expressed percentages of different aerated lung volumes. Adapted from Zhou et al. [21]

In another study including 16 patients undergoing decompressive laparotomy, different lung volumes were calculated with computed tomography at baseline, before and after decompressive laparotomy [21]. IAP increased from 12 mmHg at baseline to 25 mmHg prior to laparotomy. Total lung volume decreased from 3.2 to 2.4 L, and the percentage of atelectatic and poorly aerated lung increased. Following laparotomy, these lung changes partially reversed (Fig. 2). In these patients, laparotomy reduced IAP from 25 to 15 mmHg and improved lung volumes from 2.4 to 2.9 L [21]. Not only was the diaphragm cranially displaced but the lungs also expanded their sagittal diameter in compensation [21].

#### Key message 2: Effect of IAH on lung volumes

The presence of IAH is associated with a decrease in lung volumes, while decompressive laparotomy results in an improvement in lung volumes.

### Effect of intra-abdominal hypertension on respiratory mechanics

#### Pathophysiology

By using oesophageal catheter, the total respiratory system compliance (*C*_RS_) can be compartmentalized and chest wall (*C*_CW_) and lung compliance (*C*_L_) are derived (see below). Table 1 summarizes the factors that might affect *C*_CW_ and can be broadly divided into direct influence and indirect influence of chest wall via IAH.

#### Animal data

In pigs with IAH and healthy lungs, respiratory compliance has been shown to decline mainly due to a reduction in *C*_CW_ [15, 19, 22]. With increasing IAP, both *C*_RS_ and *C*_CW_ compliances decrease significantly [19, 22]. This decrease is more pronounced for the chest wall and shows a strong inverse correlation with IAP [22]. Previous studies in animal and human focusing on the importance of IAH showed that abdominal and subsequently chest wall compliance improves after abdominal decompression [16, 17].

#### Human data

In humans, IAH also appears to impair respiratory system compliance mainly through a reduction of chest wall compliance. Ranieri et al. [16] assessed respiratory mechanics in 18 patients with acute respiratory distress syndrome (ARDS). Half of these patients required major abdominal surgery. Before surgery, these patients had a smaller *C*_RS_ and *C*_CW_ in comparison to the patients that did not require abdominal surgery and their respiratory mechanics partially improved after decompressive laparotomy.

Gattinoni et al. [23] equally assessed respiratory mechanics in 9 patients with extrapulmonary ARDS and 12 patients with pulmonary ARDS. The patients with extrapulmonary ARDS had higher IAP levels and smaller *C*_CW_.

Despite IAH being a frequent cause of reduced chest wall compliance, it is still neglected by critical care physicians [5].

#### Key message 3: Effect of IAH on respiratory mechanics

The effects of IAH on respiratory function can be characterized by a decrease in chest wall compliance.

### Airway pressures and abdominal-thoracic transmission

#### Pathophysiology

IAH can increase intra-thoracic pressures and thereby affect airway pressures as well as pleural and central vascular pressures [7, 19, 21]. In this context, abdominal-thoracic transmission (ATT) describes the percentage increase in thoracic pressures for each incremental increase of IAP [22].

#### Animal data

In pigs, peak and plateau airway pressures increase proportionally with raising IAP [24, 25]. ATT for plateau pressure has been found to be between 40 and 50% [22, 26, 27]. ATT for peak airway pressure has been found to be between 38 and 62% [18, 22]. Similarly, oesophageal pressure is subject to ATT. Mainly inspiratory pleural pressure increase due to IAH with reported inspiratory pleural pressure of between 35 and 63% [19, 22]. In a pig study (*n* = 11), IAH up to 30 mmHg resulted in an ATT between 17 and 62% when looking at end-expiratory and end-inspiratory oesophageal pressures respectively [22].

**Fig. 3:**
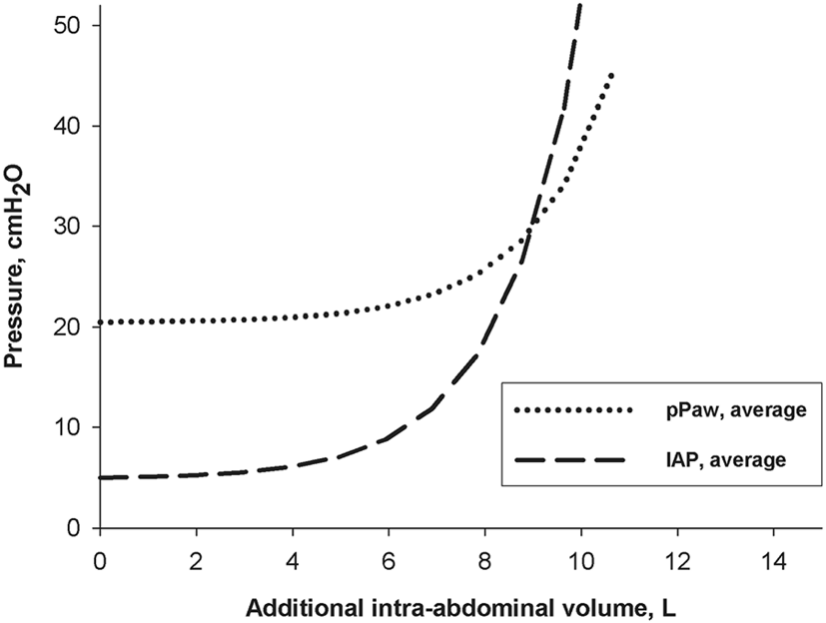
Exponential pressure–volume curves of intra-abdominal pressure (IAP) (dashed curve) and peak airway pressure (pPAW) (dotted curve) in centimetre of water in function of increasing additional intra-abdominal volume in litres derived from 7 pigs. Figure reproduced with permission from Regli et al. [26]

Increasing intra-abdominal volume increases IAP exponentially [5, 6, 26, 28]. In a pig model of IAH, increasing intra-abdominal volume has also been shown to increase peak airway pressures exponentially (Fig. 3) [26]. This exponential pressure-volume relationship is well-known from the Monroe–Kellie doctrine used in patients with intra-cranial hypertension. Similarly, in patients with IAH that already have a large amount of additional intra-abdominal volume, small changes in intra-abdominal volume can significantly affect IAP and airway pressures [5, 6].

#### Human data

In humans, ATT for plateau pressure can be estimated to be 20%. Torquato et al. [24] showed that placing 5 kg weights on the abdomen of mechanically ventilated critically ill patients increased IAP from 10.5 to 15.6 cmH_2_O and plateau airway pressures from 22.4 to 23.6 cmH_2_O). In another study, ATT for peak airway pressure was around 62% [21].

A pilot study in 14 mechanically ventilated patients with ARDS showed that the application of an abdominal Velcro belt increased IAP from 8.6 to 15.4 mmHg with a concomitant increase in alveolar plateau pressures from 18.0 to 23.3 cmH_2_O (data on file). ATT for plateau pressure was therefore 57%. These changes were paralleled by a decrease in dynamic *C*_RS_ from 37 to 28 mL/cmH_2_O. It might be important to note that the above studies were performed in the supine position yet we know that body position has a substantial influence on IAP and lung function [25].

#### Effect of abdominal-thoracic transmission on trans-pulmonary pressures

Trans-pulmonary pressures, the difference between airway and pleural pressures, and not the plateau pressures are thought to be responsible for causing ventilator-induced lung injury [29, 30]. Because IAH increases inspiratory peak airway, plateau and pleural pressures similarly, the impact on trans-pulmonary pressures is only minimal. This explains why suggested lung-protective ventilation strategies with maintaining plateau pressures below 30 cmH_2_O are difficult to apply in patients with IAH and diminished chest wall compliance.

#### Key message 4: Effect and transmission of IAH on airway pressures

In summary, in animals and humans, ATT of peak and plateau airway pressure have been reported to be between 20 and 60% [31]. In pigs, ATT of airway pressures and pleural pressures are similar. We suspect this to be the case also in humans. IAH has little influence on trans-pulmonary pressures as IAH increases both inspiratory airway and pleural pressures equally.

### Lung oedema and lymphatic drainage

#### Pathophysiology

Fluid drainage from the lungs can take place via three mechanisms: trans-pleural, via the lung hilus or transabdominal [32].

#### Animal data

Mechanical ventilation with positive pressure per se as opposed to spontaneous ventilation decreases abdominal lymphatic drainage [33]. A landmark paper by Quintel et al. [15] showed that IAH causes an increase in lung oedema in a pig model of acute lung injury (induced by oleic acid). Increasing IAP from 0 to 20 cmH_2_O changed lung oedema distribution from the dorsobasal regions to the complete lung. In line with these results, Schachtrupp et al. [34, 35] showed an increase in extravascular lung water (EVLW) and histological lung alterations at IAP levels of 30 cmH_2_O.

The effects of different ventilatory settings and increasing IAP on thoracic and abdominal lymph flow was studied in a porcine endotoxin sepsis model [36]. The study was performed in three parts, and data were collected from a total of 32 pigs. In summary, the authors found that lipopolysaccharide infusion increased IAP and abdominal (trans-diaphragmatic) lymphatic drainage, that PEEP increased IAP but impeded abdominal lymphatic drainage, that spontaneous breathing improved abdominal lymph drainage, and finally that IAH diminished abdominal lymphatic drainage [37].

#### Human data

A retrospective observational study of 123 mechanically ventilated patients found that the patients that achieved a negative fluid balance in their first week of ICU stay had lower EVLW, IAP, and C-reactive protein over albumin ratios as well as a higher 28-day survival rate [38]. This correlation between IAP, fluid balance and EVLW suggests a link between sepsis, capillary leak, fluid overload, IAH and lung oedema. This may explain why active fluid removal or so-called de-resuscitation with PAL-treatment (PEEP in cmH_2_O set at the level of IAP in mmHg, followed by hyperoncotic albumin 20% and Lasix^®^) was able to reduce cumulative fluid balance, IAP, EVLW and 28-day mortality in a retrospective matched case-control study of 57 patients with acute respiratory failure [39, 40]. Different pathologies and treatments can markedly influence the pathophysiology of the lymphatics with dramatic effects on end-organ function.

#### Key message 5: Effect of IAH on lymphatic

The presence of IAH affects lymphatic drainage between the thoracic and abdominal cavity and may play an important role in the development of oedema formation.

### Oxygenation and ventilation

#### Pathophysiology

In general, IAH results in a decreased oxygenation and an increase in hypercarbia caused by increased dead-space ventilation and shunt and ventilation perfusion mismatch.

#### Animal data

In pigs, it has been shown that IAH is associated with a redistribution of blood flow from dependent atelectatic lungs to non-dependent better ventilated lung regions thereby improving ventilation/perfusion matching [41]. This helps to explain why IAH in the context of non-injured lungs only minimally affects oxygenation in animals [18, 19, 22] and humans [42].

#### Human data

Results from a large meta-analysis on 1664 critically ill patients showed that IAH is correlated with the respiratory sequential organ failure subscore [9].

#### Key message 6: Effect of IAH on oxygenation

The presence of IAH may result in decreased oxygenation.

### Intra-abdominal hypertension and lung injury

#### Pathophysiology

It is hypothesized that IAH may result in the opening and closing of lung units and this shear stress may result in ventilator-induced lung injury.

#### Animal data

Animal studies have shown that increasing IAP during mechanical ventilation may result in cytokine release and subsequent lung injury. Rezende-Neto et al. [43] showed in a study of 50 rats that 60–90 min of IAH (IAP of 20 mmHg via insufflated intra-peritoneal air) resulted in increased plasma levels of IL-6, increased polymorphonuclear leucocytes activity in lungs as evaluated by myeloperoxidase assay and intense pulmonary inflammatory infiltration including atelectasis and alveolar oedema on lung histology. The level of applied PEEP is not mentioned. Schachtrupp et al. [44] showed in a study of 12 pigs that 24 h of IAH (IAP of 30 mmHg) also resulted in histological findings similar to those found in lung injury (interstitial and alveolar leucocytes and fibrin) but also proximal tubular and paracentral necrosis in kidneys and the liver respectively. PEEP of 2 cmH_2_O was applied. In a rat model of ARDS, IAH (15 mmHg) was associated with increased inflammation and fibrogenesis [45]. Lima et al. [46] found in a study of 20 rats that a 3-h exposure to an IAP of 15 mmHg was sufficient to cause alveolar collapse, haemorrhage, interstitial oedema, and neutrophil in infiltration in the lungs and increased lung cell apoptosis despite application of lung-protective ventilation.

#### Human data

No human data is available whether IAH is a promotor of ventilator-induced lung injury. It is likely that low trans-pulmonary pressures in the context of IAH can promote shear stress with increased repetitive opening and closing of alveoli units, even when protective tidal volume is used [47].

#### Key message 7: Effect of IAH on VILI

The presence of IAH may add to the development of VILI.

### Summary effects of IAH on respiratory function

In summary, the effects of IAH on respiratory function can be characterized by a decrease in lung volumes and chest wall compliance and an increase in airway pressures. Transmission of abdominal pressures to the thoracic cavity is estimated between 20 and 60%, but more human data is required. IAH diminishes abdominal lymphatic drainage. The presence of IAH may impair ventilation and oxygenation. Although IAH is associated with lung injury, the exact mechanism is yet not fully understood.

## Respiratory effect of IAH in the context of specific medical conditions

### Obesity

Studies have shown that obese patients with a body mass index higher than 35–40 kg/m^2^ have higher IAP values compared to non-obese patients [25, 48]. Similarly, to patients with IAH, the increased IAP values seen in obese patients will equally result in impairment in respiratory mechanics and gas exchange, and decreased lung volumes particularly during sedation, paralysis and mechanical ventilation [49]. As a consequence, the mechanical load exerted on the diaphragm is increased, especially in the supine position both during spontaneous breathing and general anaesthesia [8].

Whereas *C*_CW_ accounts in normal conditions for only 15% of the *C*_RS_, this number may increase up to 50% during patients with obesity or IAH with IAP above 20 mmHg (due to the stiffening of the chest wall) [23, 50–52]. With increasing IAP, both total *C*_RS_ and *C*_CW_ decrease significantly [19, 22]. This decrease is more pronounced for the chest wall and shows a strong inverse correlation with IAP [22]. In pigs with injured lungs, IAH has been found to decrease *C*_RS_ by decreasing not only *C*_CW_ but also *C*_L_ [15, 19]. Anaesthesia of obese patients for non-bariatric surgical procedures requires knowledge of typical comorbidities and their respective treatment options [49, 53]. A multimodal analgesia approach may be useful to reduce postoperative pulmonary complications [54].

### Acute respiratory distress syndrome

ARDS is a syndrome and not a disease. As a consequence, not all ARDS patients are the same which may be a possible explanation for some conflicting results in previous ARDS studies.

The effect of IAH on the respiratory system appears to be strongly influenced by the presence of lung injury. In pigs with injured lungs, IAH has been found to decrease *C*_RS_ by decreasing not only *C*_CW_ but also *C*_L_ [15, 55]. Furthermore, only in injured lungs, IAH has a profound effect on oxygenation [15, 55]. The decrease in *C*_L_ and oxygenation in the context of IAH and injured lungs is significant and may help understand some differences found when applying ventilation strategies in patients with IAH. This is also relevant in understanding the pathophysiologic effects of proning in patients with secondary ARDS due to IAH [56].

Ranieri et al. [16] found that patients with ARDS had different respiratory mechanics depending upon the underlying aetiology and the presence of IAH. He found that surgical patients had stiffer chest walls compared to medical patients, probably due to the increased presence of abdominal distension. Respiratory system and chest wall compliance improved after decompressive laparotomy in these patients. Unfortunately, the effect of positive end-expiratory pressure (PEEP), forced residual capacity and IAP was not measured. Mergoni and colleagues [57] studied partitioned respiratory system mechanics and showed that in a subgroup of ARDS patients in which the lower inflection point was mainly determined by *C*_CW_ that PEEP was not as effective in improving oxygenation (*C*_CW_ determined ARDS). However, PEEP was effective in ARDS patients in which the lower inflection point was determined by the *C*_L_ (*C*_L_ determined ARDS),

In contrast to this, Gattinoni et al. [23] showed that primary ARDS resulted in a decreased *C*_L_ but normal *C*_CW_ (*C*_L_ determined ARDS) while secondary ARDS presented with preserved *C*_L_ but decreased *C*_CW_ (*C*_CW_ determined ARDS), and PEEP allows to recruit lung units only in secondary but not in primary ARDS. In this study the patients with secondary ARDS had IAH as opposed to the patients with primary ARDS [23]. The results imply that the application of PEEP in pulmonary ARDS without IAH may cause over-distension of already open lung units, making these patients more prone to ventilator-induced lung injury than patients with secondary ARDS and IAH. The differences found between these two studies can in part be explained by the difference in measurement manoeuvres and techniques as well as the assumptions used [23, 57].

The same phenomenon may be responsible for the change in respiratory mechanics seen in morbidly obese patients [52]. Therefore, measuring IAP may provide an easy bedside method to estimate altered chest wall mechanics and avert the need to measure oesophageal pressure (see below). IAP also influences the shape of the pressure–volume curve (with downward flattening and rightward shifting) of the total respiratory system and the chest wall while the lung mechanics remain unaffected [15].

In summary, the presence of lung injury appears to strongly influence how IAH affects respiratory mechanics and oxygenation. Ideally, IAP is measured in ARDS patients enrolled in clinical trials to account for any influence of potential coexisting IAH.

### Polycompartment syndrome

The abdominal compartment has unique effects because it is anatomically situated “up-stream” from the extremities and “down-stream” from the thorax and the cranium [7]. Therefore, IAH may influence the physiology and pathophysiology of each of these other compartments. Because the abdomen plays a major role in the interactions between different compartments, IAP affects portal and hepatic vein pressure hence facilitating blood shunting away from the lungs, sometimes referred to as hepato-abdominal-pulmonary syndrome [7]. Similarly, IAP has been identified as the missing link triggering renal failure (via increased renal vein pressures) in patients with chronic congestive heart disease, referred to as cardio-abdominal-renal syndrome [58]. Likewise, deteriorating kidney function in patients with liver cirrhosis is called hepato-abdominal-renal syndrome.

## Practical implications at the bedside and respiratory management in intra-abdominal hypertension

Table 3 lists suggested ventilation strategies for patients with IAH and ARDS.

**Table 3:** Suggested ventilation strategies depending on the presence of ARDS and IAH

	Normal	ARDS	IAH	IAH and respiratory failure
Tidal volume	6 to 8 mL/kg PBW may be beneficial	Recommended 4–8 mL/kg PBW (Grade 1B) [123, 124]	6 to 8 mL/kg PBW may be beneficial	4–8 mL/kg PBW may be beneficial
Inspiratory plateau pressure	< 20 cmH_2_O	Recommended < 30 cmH_2_O to reduce risk of alveolar over-distension (Grade 1B) [123]	Higher airway pressures may be acceptable and may arise due to reduced chest wall compliance. Corrected target plateau pressure = target plateau pressure − 7 + IAP (mmHg) * 0.7	Higher airway pressures may be acceptable and may arise due to reduced chest wall compliance. Corrected target plateau pressure = target plateau pressure − 7 + (mmHg) * 0.7
Driving pressure	< 14 cmH_2_O	< 14 cmH_2_O (Grade 2B) [84]	< 14 cmH_2_O	< 14 cmH_2_O
Inspiratory plateau trans-pulmonary pressure	< 15 cmH_2_O is reasonable	< 25 cmH_2_O is reasonable [29]	< 25 cmH_2_O is reasonable	< 25 cmH_2_O may be a reasonable target
PEEP	5 in cmH_2_O	Higher PEEP levels in moderate to severe ARDS improves survival rate (Grade 2B) [123]. We suggest 5–10 cmH_2_O in mild to moderate ARDS and 10–15 in moderate to severe ARDS	Higher PEEP levels may reduce atelectasis and atelectrauma. We suggest not to exceed 15 cmH_2_O	Higher than usual PEEP levels may be required to improve oxygenation and respiratory mechanics. We suggest not to exceed 15 cmH_2_O
PEEP titration	We suggest avoidance of excessive driving pressure	Optimal respiratory compliance, i.e. lowest driving pressure during constant protective tidal volume. Oesophageal pressure guided is a reasonable alternative	Optimal respiratory compliance, i.e. lowest driving pressure during constant protective tidal volume. We suggest PEEP in cmH_2_O = IAP in mmHg	Optimal respiratory compliance, i.e. lowest driving pressure during constant protective tidal volume. Oesophageal pressure guided is a reasonable alternative
Recruitment manoeuvre (RM)	RM not routinely recommended	RM improves oxygenation, but outcome may be worsened with RM. Best RM method is unknown [65, 123]	RM not routinely recommended	Higher airway pressures might be required for RM to be effective
Prone	Not recommended	Recommended as it improves oxygenation and survival rate in patients with ARDS (Grade 1B) [108, 123]	Not recommended	May reduce IAP and improve oxygenation Important to assure free hanging abdomen and absent IAP increase [29]
NMBA	Not recommended	Short term NMBA may be beneficial [125]	May reduce IAP [121]	May reduce IAP and/or improve oxygenation
Adjunctive therapy		Nitric oxide	Negative fluid balance	Negative fluid balance
		ECCO_2_R	Ascites drainage	Ascites drainage
		ECMO	Laparostoma [26, 121]	Laparostoma
				Nitric oxide, ECCO_2_R, ECMO

*ARDS* acute respiratory distress syndrome, *ECCO2R* extracorporeal CO_2_ removal, *ECMO* extracorporeal membrane oxygenation, *IAH* intra-abdominal hypertension, *IAP* intra-abdominal pressure, *PBW* predicted body weight, *PEEP* positive end-expiratory pressure, *RM* recruitment manoeuvre

### Measuring intra-abdominal pressure

The easiest way to assess IAP in clinical practice is by measuring bladder pressures [1, 59, 60]. The reference standard for intermittent IAP measurement is via the bladder with a maximal instillation volume of 25 mL of sterile saline and IAP should be measured at end-expiration in the supine position after ensuring that abdominal muscle contractions are absent and with the transducer zeroed at the level of the midaxillary line.

Although abdominal contractions can falsely increase IAP values, we don’t recommend increasing sedation or using neuromuscular blocking agents to improve accuracy of IAP measurements. In our clinical experience, IAP can be accurately measured in patients that either receive assisted breaths during mechanical ventilation or don’t have any respiratory support. It is important however to sufficiently extend the observation period in order to capture the lowest end-expiratory pressure during which abdominal contractions are clinically (visible and palpable) absent.

### Measuring oesophageal pressure

From dividing the tidal volume by the difference between plateau pressure and positive end-expiratory pressure (driving pressure), *C*_RS_ can be calculated. By using an oesophageal catheter (as surrogate for intra-thoracic pressure), *C*_CW_ and *C*_L_ can also be estimated. In addition, trans-pulmonary pressures is the difference between airway and pleural pressures and is thought to be the main determinant in causing ventilator-induced lung injury [29, 30].

However, measuring oesophageal pressure is not easy due to some practical problems at the bedside [8, 61]. It requires a small air-filled balloon that can transmit the oesophageal pressure via catheter to a pressure transducer. Newer oesophageal catheters are integrated in to a nasogastric feeding tube. The catheter is first placed into the stomach, then withdrawn back into the oesophagus and requires an occlusion test to confirm the correct placement. In addition, the catheters are prone to under-and overestimate oesophageal pressures if to little or too much air is instilled. In some patients correct placement is not possible.

### Recruitment manoeuvres

A recruitment manoeuvre (RM) uses a dynamic and transient increase in the trans-pulmonary pressure to open non-aerated or poorly aerated lung areas [62]. The benefit of improved oxygenation may be offset by a potential epithelial and endothelial cell damage and increased alveolar-capillary permeability [63, 64]. Furthermore, in a recent large randomized controlled trial patients with ARDS receiving RM and PEEP titrated to their best respiratory system compliance (lowest driving pressure during constant protective tidal volumes) as opposed to no RM and low PEEP to had a reduced survival rate [65].

Frequently a fast RM manoeuvre is performed by applying 40 cmH_2_O inspiratory pressure for 40 s (40-by-40 manoeuvre) [66, 67].

However, in recent years, following the results of several experimental studies [67–70] and clinical trials [66, 71] slow RM are preferred over fast RM since this is associated with improved oxygenation, less inflammation, and improved haemodynamical instability.

In principle, slow RM are performed by gradually increasing and then decreasing PEEP and/or tidal volumes until plateau pressures of between 40 and 50 cmH_2_O are achieved while up keeping tidal ventilation [66, 72].

It is estimated that a trans-pulmonary opening pressure equal to 30 cmH_2_O is required to open atelectasis. In the setting of IAH with altered *C*_L_/*C*_RS_ ratio from 0.85 to 0.5 the resulting trans-pulmonary pressure during a 40-by-40 recruitment manoeuvre may only be 20 cmH_2_O, hence the alveolar units with long time constants would remain collapsed [61]. Therefore, in the setting of IAH, higher opening pressures closer to may be required [29]. The rational for adding IAP in cmH_2_O/2 is due to the ATT being around 50% [18, 19, 22, 24, 27, 55]. However, applying higher inspiratory opening pressures during a RM is more likely to cause haemodynamic compromise. Therefore, RM should only if at all be applied in haemodynamically stable patients (e.g. not preload dependent) and their blood pressure needs to be closely monitored if RM is applied.

In summary, no studies have been performed in patients with IAH assessing different RM methods. We therefore suggest to use RM manoeuvres with caution in patients with IAH.

### Ventilator settings during lung-protective ventilation in patients with IAH

It is generally recommended to provide protective lung ventilation in patients with IAH and ARDS [29]. Even in patients with non-injured lungs, protective lung ventilation is becoming more frequently applied as this has been associated with less inflammation and fewer pulmonary complications [73].

#### Tidal volumes

There are no studies assessing optimal tidal volumes in patients with IAH. In a rat model of ARDS and IAH (15 mmHg), Santos et al. [45] found that 10 mL/kg as opposed to 6 mL/kg was associated with reduced inflammation in the subgroup with extrapulmonary ARDS and increased inflammation in the subgroup with pulmonary ARDS. However, there are many studies demonstrating high tidal volumes in patients with ARDS worsens outcome [74].

The perioperative use of lower tidal volumes (6–8 mL/kg of predicted body weight) plus the application of PEEP as opposed to the use of higher tidal volumes (10–12 mL/kg of predicted body weight) and no PEEP is associated with reduced respiratory complications in patients undergoing major abdominal surgery [73]. However, other studies and meta-analysis showed that, even in clinical conditions characterized by higher IAH, the reduction in tidal volume and not higher PEEP per se was associated with improved postoperative outcome [75, 76].

In the absence of any evidence regarding optimal tidal volumes in patients with IAH, it is not unreasonable to apply lung-protective ventilation with low tidal volume of 6–8 mL/kg of predicted body weight also in all patients with IAH and particularly in patients with IAH and lung injury.

#### Airway pressures

Lung-protective ventilation implies opening the lungs with a RM (appropriate high alveolar pressures) and keeping the lungs open (with appropriate PEEP setting) [77]. The altered lung mechanics in the context of IAH may require higher than usual pressures to open airways and keep airways open exceeding those set out in current guidelines [55]. Lung-protective ventilation is recommended using an upper limit goal for plateau pressures of 30 cmH_2_O in patients with severe ARDS [78]. These recommendations don’t take IAP into account. The rationale behind limiting the plateau pressure is to avoid increased trans-pulmonary pressures, alveolar over-distension and ultimately ventilator-induced lung injury [79].

Ideally, to avoid alveolar over-distension one would measure oesophageal pressure in critically ill patients and aim for inspiratory trans-pulmonary pressures < 25 cmH_2_O respectively [29, 61, 79, 80]. However, oesophageal pressure measurements are not easy to perform and challenging [61, 81].

As stated above, IAH is associated with raised airway pressures. ATT is around 50% affecting oesophageal and airway pressures similarly [19, 22, 31]. This means that in the context of IAH, when applying appropriate PEEP levels and lung-protective tidal volumes of 6 mL/kg PBW, plateau pressure can exceed the recommended 30 cmH_2_O without necessary affecting trans-pulmonary pressures.

It follows that in the presence of IAH airway pressures could be corrected by using ATT. In critically ill subjects IAP is on average 10 mmHg = 13.6 cmH_2_O [4, 9, 82] and we can hypothesize that half of this pressure is normally transmitted in the presence of normal chest wall. Thus, corrected plateau pressure target in cmH_2_O may be calculated as follows: plateau pressure target in cmH_2_O + [(IAP in mmHg * 1.36) − 13.36 (normal IAP in critical patients)]/2 or simplified: plateau pressure target − 7 + 0.7 * IAP in mmHg.

For example, for a target plateau pressure of 30 cmH_2_O and an IAP of 20 mmHg the corrected target plateau pressure would be: 30 − 7 + 0.7 * 20 * = 37 cmH_2_O. In the absence of IAH, plateau pressures target would not require any correction. For example, for target plateau pressure of 30 and IAP 10 mmHg the corrected plateau pressure target would be 23 + 10 * 0.7 = 30 cmH_2_O.

In summary, although higher airway pressures might be acceptable in ARDS patients with IAH we are lacking any supporting clinical data to make any recommendations.

#### Driving pressure

In more recent years, driving pressure (pressure difference between plateau airway pressure and PEEP) as a measure of protective lung ventilation has gained more attention.

Driving pressure appears to be helpful to optimize tidal volume and to avoid excessive dynamic strain. Using data from nine randomized trials and a total of 3562 patients with ARDS, it has been demonstrated that the driving pressure has a greater influence on mortality than plateau airway pressure, tidal volume or PEEP [83]. A large international observational study of 29,144 ventilated patients found that a driving pressure of less than 14 cmH_2_O is associated with improved hospital survival in patients with ARDS [84]. In obese ARDS patients however, an increased driving pressure was not associated with an increased mortality [85]. No studies specifically assess the effect of different driving pressure on outcome in patients with IAH or in obese patients with IAH. However, it is not unreasonable to apply a driving pressure of less than 14 cmH_2_O in patients with IAH.

Driving pressure may also be useful in titrating PEEP and has been tested with promising physiological results in obese patients undergoing general anaesthesia [86, 87] and in obese patients with ARDS [88, 89]. An individual patient data meta-analysis showed that obese patients undergoing surgery receiving higher PEEP levels that resulted in an increased driving pressure had more postoperative pulmonary complications [90]. There is currently insufficient data to suggest using driving pressure to titrate PEEP might translate in improved survival of patients with IAH or obesity with or without ARDS. Thus, in our opinion driving pressure should be used to avoid excessive PEEP and not to “optimize” (increase) PEEP.

#### PEEP

To date, the best PEEP to be used in the setting of IAH remains unknown [91]. As stated above, in the setting of IAH the lung will collapse at higher closing pressures during expiration. There remains the fear that in the context of IAH, increased atelectrauma (also referred to as atelectotrauma) due to increased atelectasis formation and an insufficient PEEP may contribute to the lung injury found in the presence of IAH [8, 47, 92]. Therefore, higher PEEP levels might be required to keep lungs open and reduce lung injury in the context of IAH. In contrast to this, higher PEEP levels may not only have negative haemodynamic effects but also cause lung injury if alveolar over-distension occurs [44, 92].

Different animal studies have examined different levels of PEEP in the setting of IAH. A first study was conducted in 13 pigs with healthy lungs and IAH was created with an inflatable balloon, the PEEP levels (5, 8, 12 and 15 cmH_2_O) were unmatched to the level of IAP [18]. The conclusions were that commonly applied PEEP levels, set below the IAP level may not prevent end-expiratory lung volume decline. Noteworthy was that IAP reached 18 mmHg or thus 25 cmH_2_O, while PEEP was only set up to a maximum of 15 cmH_2_O. In a second study, conducted in 9 pigs with healthy lungs, IAH was again created with an inflatable balloon, the PEEP levels were now matched for IAP [19]. The authors found preservation of end-expiratory lung volume without improvement in arterial oxygen tension but with a reduction in CO. In a third study, conducted in 8 pigs with lung injury induced by saline lavage and IAH created with CO_2_ insufflation up to 20 mmHg, the PEEP levels (27 cmH_2_O) were matched for IAP [93]. The major findings during PEEP application were lower inflection point, improved compliance, decreased alveolar-arterial gradient and less shunt. In a fourth animal study in 9 pigs, IAH induced by an inflatable balloon was combined with oleic acid-induced lung injury, and PEEP levels were matched to IAP [55]. The authors found better end-expiratory lung volumes, lower shunt fraction, lower dead space and a better oxygenation.

There are only few clinical studies. Krebs et al. [94] examined different levels of PEEP in 20 patients with ARDS, ten had normal (IAP of 8 mmHg) and 10 had grade II IAH (IAP of 16 mmHg). No difference was found between the groups at baseline. This might explain why no differences were found between the groups regarding the effect of higher levels of PEEP on lung mechanics or oxygenation.

In a different study Krebs et al. [95] examined two methods of PEEP titrated in 13 patients with moderate to severe ARDS. They found that in patients with IAH, the best PEEP set according to the best compliance of the respiratory system, is not always associated with positive end-expiratory trans-pulmonary pressure.

Gattinoni et al. [23] applied different levels of PEEP (5, 10, 15, and 20 cmH_2_O) in patients with ARDS. The patients with extrapulmonary ARDS had IAH (IAP of 16 mmHg) and PEEP improved *C*_RS_ due to a reduction in *C*_CW_. In contrast, the patients with pulmonary ARDS had normal IAP (4 mmHg) and PEEP worsened *C*_RS_ due to an increase in *C*_L_.

Talmor et al. [29, 96] found that IAP (measured via the stomach) and oesophageal pressure (measured via an oesophageal balloon) closely correlated. Therefore, not only opening pressures but also closing pressures are increased during IAH and as such higher PEEP levels may be required to prevent end-expiratory lung collapse.

In a pilot study of 15 patients with IAH (IAP of 17 mmHg), different levels of PEEP were applied that were matched to the level of IAP [97]. In contrast to PEEP = 50% of IAP, PEEP = 100% of IAP (both parameters measured in equal units) was not well tolerated due to hypoxaemia, hypotension or endotracheal cuff leak.

It is difficult to draw any conclusions from these experimental and clinical studies. In principle, it makes sense to apply higher PEEP levels in the context of IAH. Furthermore, it is appealing to apply an easy to use bedside formula for setting the PEEP level in patients with IAH, e.g. PEEP (cmH_2_O) to be set to the level of IAP (mmHg).

There is a concern that increasing PEEP can increase IAP. Many published studies found PEEP to have only minimal influence on IAP (increasing PEEP from as low as 0 to as high as 15 cmH_2_O and average IAP increase of 1 mmHg) [25]. In contrast to this, Verzilli et al. [98] examined the effect of raising PEEP from 0 to 12 cmH_2_O in 30 patients with ARDS and found that IAP increased predominantly in patients with IAH (i.e. IAP increased from 15 to 20 cmH_2_O).

In summary, while PEEP can counteract the negative effects of IAH on lung volume and chest wall compliance, there is no evidence that a certain PEEP level improves outcome in patients with IAH. In the absence of any evidence we recommend to set the PEEP according to the best respiratory system compliance [92].

#### IAH mode of ventilation and assisted breathing

Assisted breathing is the most common type of ventilation in critically ill patients [99] and even in patients with ARDS [84]. The potential advantages of assisted breathing include less need of sedation and haemodynamic impairment, minimal muscular atrophy, better lymph drainage and regional organ perfusion [36, 100].

Little is known about the optimal ventilation mode to be applied in patients with IAH but some experimental data exist. Several experimental evidences of ARDS without IAH showed reduced lung injury during assisted ventilation [101–104]. Recent results from an animal experiment suggests that assisted ventilation might be associated with improved oxygenation and less lung injury and inflammation in mild to moderate (extrapulmonary) ARDS in the presence of IAH (15 mmHg) [105]. This was likely due to reduced atelectasis and more homogeneous distribution of regional ventilation. However, other experimental evidence reported that addition of unsupported spontaneous breaths to BiPAP did not improve haemodynamic and respiratory function and caused greater histopathologic damage to the lungs, in the presence of severe IAH [106]. The difference in these results may be due to the amount of inspiratory effort reached during spontaneous breathing and/or different modalities of ventilation.

In conclusion, we suggest a cautious use of assisted ventilation in patients, especially if in the presence of severe IAH.

#### Prone and other positioning

Prone position improves respiratory mechanics, oxygenation and reduces over-distension [107]. Prone ventilation has been shown to improve outcome in patients with severe ARDS [108]. Placing ARDS patients in the prone or upright position does not result in univocal beneficial effects on respiratory mechanics and oxygenation parameters [52].

In the setting of IAH, there seems to be some merit by suspending and offloading the abdomen during prone ventilation. Mure et al. [109] demonstrated in an interesting animal model that the prone position improves pulmonary gas exchange to a greater degree in the presence of IAH as shown by increases in PaO_2_ and decreases in ventilation perfusion heterogeneity. The observed decrease in IAP (estimated via gastric pressure), resulting in a concomitant decrease in pleural pressure in the prone position may be a possible explanation for these observations, hence facilitating regional ventilation in the dependent lung zones near the diaphragm.

In a recent experimental study in 12 pigs that underwent pulmonary saline lavage and injurious ventilation to simulate ARDS, the authors showed that prone position and PEEP independently improved lung compliance without interaction [110]. As expected, IAH (15 mmHg) increased the PEEP needed for the best lung compliance. However, best PEEP was not significantly different between prone (12.8 ± 2.4 cmH_2_O) and supine (11.0 ± 4.2 cmH_2_O) positions when targeting lung compliance.

De Jong et al. [111] successfully applied prone positioning in obese and non-obese patients with ARDS. In obese patient, oxygenation improved significantly more than in non-obese patients. Although not measured, these obese patients would likely have had higher IAP levels.

Placing patients with ARDS in the prone position either does not change or only has mild influence on IAP levels with more pronounced effects in patients with IAH [25, 112]. For example, Jozwiak et al. [112] found a mild increase in IAP from 15 to 18 mmHg when patients with ARDS were proned.

The use of chest and pelvic suspension has a large influence on IAP pressures [113]. The pressure exerted by the chest suspension will result in a decreased *C*_CW_, while the suspension at the level of the symphysis pubis will ensure a free suspended abdomen and thereby limiting transmission of IAP towards the dorsobasal lung regions and diaphragm. This decreases IAP and improves abdominal compliance and reduces atelectasis via dorsobasal recruitment. The theoretical benefits of proning a patient with IAH need to be outweighed against the practical risks (e.g. patients with an open abdomen).

**Fig. 4:**
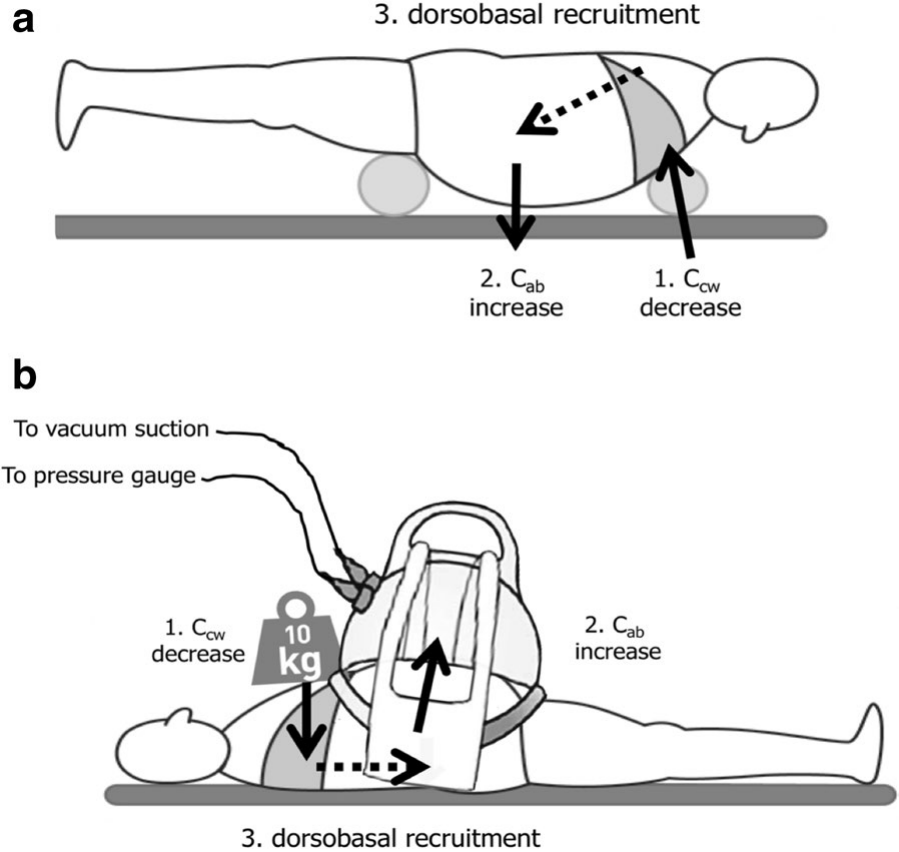
Effects of positioning on chest and abdominal wall compliance. **a** Effects of prone positioning with abdominal suspension on chest and abdominal wall compliance. The suspension placed under the chest will reduce chest wall compliance (1) while the abdominal suspension placed at the level of the symphysis will exert a gravitational effect that will increase abdominal wall compliance (2). This will result in recruitment of dorsobasal lung regions (3). **b** Effects of supine positioning in combination with weight placed on the chest and vacuum bell on the abdomen. The weight placed on the chest will reduce chest wall compliance (1) while the abdominal vacuum bell will increase abdominal wall compliance (2). This will result in recruitment of dorsobasal lung regions (3)

Interestingly, weightlessness appears to be beneficial in the setting of IAH [114]. The combination of a weight placed on the chest with a vacuum shell placed on the abdomen has similar effects to that of weightlessness with reducing *C*_CW_ and improving abdominal compliance (Fig. 4).

In summary, prone position appears to not increase IAP and likely improve oxygenation in patients with respiratory failure and IAH. However, further studies are required to compare the effectiveness on outcome of these different approaches in patients with IAH and respiratory failure.

## Potential haemodynamic compromise in patients with IAH and the application of PEEP

### Individual cardiovascular effect of IAP and PEEP

The individual effect of IAH as well as high PEEP on the cardiovascular system is well described [115–118]. Both IAH and high PEEP are associated with a reduced cardiac output. IAH decreases venous return mainly by abdominal compression of the inferior vena cava but central vascular filling pressures like central venous pressure and pulmonary artery occlusion pressure are elevated. Intra-thoracic pressure via ATT is elevated thereby raising right ventricular afterload. Left ventricular afterload is increased due to a direct compression of the abdominal capillary vessels and via an activation of the renin–angiotensin–aldosterone pathway. It is thought that in patients with IAH, cardiac output is mainly influenced by afterload [116].

PEEP exerts its cardiovascular influence by increasing intra-thoracic pressures [117]. Right and left ventricular venous return is reduced, right ventricular afterload is increased but left ventricular afterload is decreased.

### Combined cardiovascular effect of IAP and PEEP

Not much studies have assessed the combined haemodynamic effect of both IAP and PEEP. Both IAP and PEEP synergistically decrease increase right ventricular afterload. In theory, IAP and PEEP have two possible antagonistic interactions. Firstly, left ventricular afterload is increased by IAH but decreased by high PEEP levels.

Secondly, venous return from inferior vena cava to the right atrium is largely determined by the right atrial pressure (RAP) over IAP gradient [119].

In the absence of IAH (RAP > IAP), increasing IAP levels can increase venous return and improve cardiac output by redistribution of blood from the abdominal to the thoracic compartment. In the presence of IAH however (RAP < IAP), venous return and cardiac output are reduced. It is possible that increasing PEEP in the presence of IAH might favourably change the RAP over IAP gradient (RAP > IAP) and thereby improving both venous return and cardiac output.

There are only a few animal and human studies examining combined haemodynamic effect of IAH and PEEP. In animal studies PEEP (ranged of 4–22 mmHg) had a stronger negative impact on cardiac output than IAP (range of up to 26 mmHg) [18, 19, 55]. PEEP that was adjusted to half the IAP (PEEP = 50% of IAP) did not significantly reduce cardiac output in contrast to PEEP that was fully adjusted to IAP.

Krebs et al. [94] applied different PEEP levels of up to 15 mmHg (20 cmH_2_O) in 20 patients with ARDS but did not find any cardiovascular differences between the patients with and without IAH.

In a pilot study of 15 patients with IAH but healthy lungs, different PEEP levels were applied and no difference in blood pressure or heart rate was found probably due to small sample size [97].

In 8 volunteers with inflated medical anti-shock trousers (IAP not measured), additional PEEP of 10 cmH_2_O was applied and echocardiography was performed [120]. It was concluded that the increase in left ventricular afterload induced by medical anti-shock trousers inflation may be counteracted by the use of a PEEP.

In summary, limited experimental and clinical data suggest that the negative haemodynamic effect of PEEP is to some degree counteracted in patients with IAH. However, it is difficult to draw any conclusions from above studies and the clinician should be cautious when applying higher PEEP levels in patients with IAH as cardiovascular response to higher PEEP levels is difficult to predict.

## Medical management of intra-abdominal hypertension

**Fig. 5:**
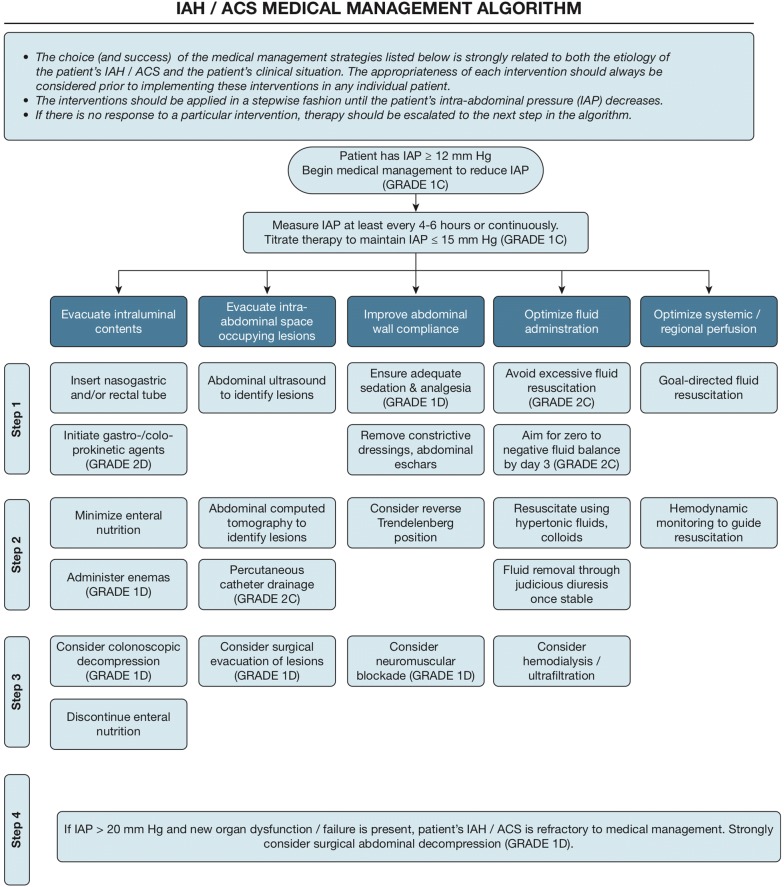
WSACS 2013 Intra-Abdominal Hypertension/Abdominal Compartment Syndrome Medical Management Algorithm. Quality of evidence for each recommendation and strength of recommendation is rated along a four-point ordinal scale in accordance with Grading of Recommendations Assessment, Development and Evaluation (GRADE) guidelines (http://www.gradeworkinggroup.org), in which each evidence grade is symbolized by a letter from D to A: very low (D), low (C), moderate (B), and high (A) and strength of recommendation is given by a number: strong (1) and weak (2). *ACS* abdominal compartment syndrome, *IAH* intra-abdominal hypertension, *IAP* intra-abdominal pressure. ©Copyright by WSACS, the Abdominal Compartment Society (http://www.wsacs.org). Figure reproduced and adapted with permission from Kirkpatrick et al. [1]

Medical management strategies for raised IAP may be divided into five categories according to their proposed mechanism of action. First, improvement of abdominal wall compliance (sedation and analgesia, neuromuscular blockade, epidural anaesthesia and body positioning changes); second, evacuation of intra-luminal contents (nasogastric or rectal decompression and use of prokinetic agents); third, drainage of intra-abdominal fluid collections (paracentesis or percutaneous catheter drainage); fourth, avoidance of excessive fluid resuscitation and correction of a positive patient fluid balance (with judicious use of fluids, e.g. rather hypertonic solutions instead of crystalloids); and fifth, organ support (respiratory and cardiovascular monitoring as outlined above) [1, 121, 122]. It would be beyond the scope of this review to discuss the different medical management strategies into detail. An overview of the WSACS IAH/ abdominal compartment syndrome medical management algorithm (and the associated GRADES of recommendations) is shown in Fig. 5. Specific to patients with IAH requiring mechanical ventilation, it is worth noting that small reductions in intra-abdominal volume can significantly improve airway pressure and IAP [26].

Monitoring extravascular lung water and pulmonary vascular permeability (calculated with trans-pulmonary thermodilution and defined as EVLW divided by pulmonary blood volume) can provide useful additional information. Deep sedation with a short course of neuromuscular blocking agents may be useful in selected patients or as a bridge towards decompressive laparotomy.

## Conclusions

Although considerable progress has been made over the past decades, some important questions remain relating to the optimal ventilation management in patients with IAH. When looking after patients with IAH and ARDS requiring mechanical ventilation, an important first step is to measure IAP and aim to reduce IAP in order to reduce airway pressures keeping in mind that small reductions in intra-abdominal volume can significantly reduce IAP and airway pressures [26].

Although challenging, the measurement of oesophageal pressure as surrogate for intra-thoracic pressure can provide trans-pulmonary pressures that can help guide ventilation [92]. It is of note that IAH can lead to the polycompartment syndrome with the associated interactions between different compartmental pressures [7]. Within this respect, one should avoid head of bed elevation above 45° in patients with high body mass index as this is associated with increase in IAP.

During lung-protective ventilation, we recommend the application of protective lung ventilation with low tidal volumes of 6–8 mL/kg and maximum driving pressure of 15 cmH_2_O. Higher than recommended plateau pressures of 30 cmH_2_O might be required in the setting of IAH. Taking normal IAP of 10 mmHg and ATT of around 50% into account, 23 cmH_2_O + 0.7 * IAP in mmHg might be an appropriate upper limit of plateau pressure.

In addition, in patients with IAH, higher PEEP levels might be required to prevent end-expiratory lung collapse. However, the best PEEP in the setting of IAH is still unknown. Pressure-volume loops or the use of oesophageal pressure might be useful to determine the best PEEP in patients with IAH. Knowing that the ATT is around 50%, it may be appropriate to set PEEP (cmH_2_O) equal to 50% of IAP in cmH_2_O. In the absence of any evidence, we recommend to set the PEEP according to the best *C*_RS_.

Anti-Trendelenburg or prone position with abdominal suspension may have beneficial effects on respiratory mechanics in patients with IAH. Monitoring the respiratory function and adapting the ventilator settings accordingly during anaesthesia and critical care is of great importance.

With our improved understanding of the pathophysiology and epidemiology, future randomized studies should be focused on defining whether targeted or multifaceted medical (and minimally invasive surgical) interventions aimed at reducing IAP and improving abdominal compliance will ultimately improve outcomes in patients with IAH and abdominal compartment syndrome.

## Abbreviations

ARDS: acute respiratory distress syndrome
ATT: abdominal-thoracic transmission
*C*_RS_: total respiratory system compliance
*C*_CW_: chest wall compliance
*C*_L_: lung compliance
EVLW: extravascular lung water
IAH: intra-abdominal hypertension
IAP: intra-abdominal pressure
ICU: intensive care unit
PEEP: positive end-expiratory pressure
RAP: right atrial pressure
RM: recruitment manoeuvre
